# Seven Good Rules for Preserving the Eye-Sight

**Published:** 1878-09

**Authors:** 


Seven Good Rules for Preserving the Eye-Sight.—Dr. H. C. Angell, in his little book on "How to take Care of our Eyes," recently published in Boston, gives the following rules to be carefully observed by all persons who have a tendency to weakness of sight, or who experience unusual fatigue of the eyes in reading or other occupation requiring close use of the eyes:

1. Cease to use the eyes for the time being, and look away from the work, when sight becomes in the least painful, blurred or indistinct. After perfect rest for a moment or longer, work may be resumed, to be discontinued as before when the eyes feel again fatigued.

2. See that the light is sufficient, and that it falls properly upon your work. Never sit facing it. It is best that the light should fall upon the work from above and behind; failing in this, it may fall from the side. Never use the eyes at twilight. Any artificial light for the evening is good, if it is brilliant enough and steady. When artificial light is at all painful, it is safer to read or write only during the day.

3. Never read in the horse or steam cars. It requires too great an exertion of the accommodative power to keep the eyes fixed on the letters.

4. Never read when lying down; it is too fatiguing for the accommodative power. Many a tedious case of weak sight has been traced to the pernicions habit of reading in bed after retiring for the night.

5. Do not read much during convalescence from illness. Before the muscular system generally has quite recovered its healthy tone, we ought not to expect the muscles of accommodation to bear the continuous use to which they are subjected in reading or writing. We can not be sure that the delicate muscles of the eye are in a condition to be used until the muscles of the leg and the arm have regained their strength and firmness.

6. The general health should be maintained by a good diet, sufficient sleep, air, exercise, amusement, and a proper restriction of hard work.

7. Take plenty of sleep. It is a sovereign balm for those who suffer from weak sight. Retire early, and avoid the painful evening lights. Ten hours' sleep for delicate eyes is better than eight.—*Boston Journal of Chemistry.*

